# High attrition among HIV-infected patients with advanced disease treated in an intermediary referral center in Maputo, Mozambique

**DOI:** 10.3402/gha.v7.23758

**Published:** 2014-04-08

**Authors:** Lucas Molfino, Ajay M. V. Kumar, Petros Isaakidis, Rafael Van den Bergh, Mohamed Khogali, Sven G. Hinderaker, Alice Magaia, Sheila Lobo, Celeste Gracia Edwards, Jan Walter

**Affiliations:** 1 Médecins Sans Frontières, Maputo, Mozambique; 2 International Union Against Tuberculosis and Lung Disease, South-East Asia Regional Office, New Delhi, India; 3 Médecins Sans Frontières (MSF), Operational Research Unit, Luxembourg City, Luxembourg; 4 Centre for International Health, University of Bergen, Bergen, Norway; 5 Direcçao de Saude da Cidade de Maputo (DSCM), Ministry of Health, Maputo, Mozambique

**Keywords:** operational research, retention in care, resource-limited settings, attrition, HIV referral center

## Abstract

**Background:**

In Mozambique, antiretroviral therapy (ART) scale-up has been successfully implemented. However, attrition in care remains a major programmatic challenge. In 2009, an intermediary-level HIV referral center was created in Maputo to ensure access to specialized care for HIV-infected patients with complications (advanced clinical-immunological stage, Kaposi sarcoma, or suspected ART failure).

**Objective:**

To determine the attrition from care and to identify risk factors that lead to high attrition among patients referred to an intermediary-level HIV referral center.

**Design:**

This was a retrospective cohort study from 2009 to 2011.

**Results:**

A total of 1,657 patients were enrolled, 847 (51%) were men, the mean age was 36 years (standard deviation: 11), the mean CD4 count was 27 cells/µl (interquartile range: 11–44), and one-third were severely malnourished. The main reasons for referral were advanced clinical stages (WHO stages 3 and 4, and CD4 count <50 cells/µl) in 70% of the cases, and 19% had Kaposi sarcoma. The overall attrition rate was 28.7 per 100 person-years (PYs) – the mortality rate was 5.0 (95% confidence interval [CI]: 4.2–5.9) per 100 PYs, and the loss-to-follow-up rate was 23.7 (95% CI: 21.9–25.6) per 100 PYs. There were 793 attritions – 137 deaths and 656 lost to follow-up (LTFU); 77% of all attrition happened within the first year. The factors independently associated with attrition were male sex (adjusted hazard ratio [aHR]: 1.15, 95% CI: 1.0–1.3), low body mass index (aHR: 1.51, 95% CI: 1.2–1.8), WHO clinical stage 3 or 4 (aHR: 1.30, 95% CI: 1.0–1.6; and aHR: 1.91, 95% CI: 1.4–2.5), later year of enrollment (aHR 1.61, 95% CI 1.3–1.9), and ‘being already on ART’ at enrollment (aHR 13.71, 95% CI 11.4–16.4).

**Conclusions:**

Attrition rates among HIV-infected patients enrolled in an intermediary referral center were high, mainly related to advanced stage of clinical disease. Measures are required to address this, including innovative strategies for HIV-testing uptake, earlier ART initiation and nutritional supplementation, and special attention to men and those who are already on ART at enrolment. Qualitative research is required to understand the reasons for being LTFU and design informed evidence-based interventions.

In Mozambique, a resource-poor country in southern Africa, HIV infection continues to be a major public health problem. An estimated 1.6 million people are living with HIV/AIDS which remains the leading cause of death in adults aged 15–49 years in the country (1, 2). Over the last 10 years, the national HIV/AIDS program has made remarkable efforts to combat the HIV epidemic, with a rapid decentralization of services to lower-level health facilities across the country in order to scale up access to antiretroviral treatment (ART).

While the success of the ART scale-up has been well documented globally, attrition (death, lost to follow-up [LTFU], and stopping ART) among patients in care remains a major programmatic challenge (3, 4). Previous studies from Mozambique have reported high rates of attrition before and after initiation of ART, with most patient attrition occurring within the first year after ART initiation, and rates have been found to be higher in peripheral health clinics as compared to vertically implemented urban HIV clinics (5, 6). Possible reasons for this high attrition could be related to the lack of capacity to manage complicated cases of HIV and the lack of secondary-level referral centers in some health districts for the management of such cases (7).

In order to bridge the gap, Médecins Sans Frontières (MSF), in collaboration with the Ministry of Health of Mozambique, created an intermediary-level HIV care center (the Referral Centre of Alto Mae [CRAM]) in Chamanculo Health District (CHD) in Maputo City to ensure access to specialized care for patients living with HIV who had complications (including those with low CD4 counts, with advanced clinical stage, with Kaposi sarcoma, and/or in need of second-line ART). Thus, the patient cohort in CRAM was already at a higher risk of attrition. To add to the concern, annual program reports indicated an increasing trend of attrition from 24% in 2009 to 32% in 2011 (8). Hence, a good understanding of the pattern and causes of attrition is needed to improve program performance, contribute to patient survival, and reduce the risk of further HIV transmission in the community (9). In this study, we aimed to describe the overall attrition rates among patients living with HIV and enrolled in CRAM, discuss the timing of such attrition, and determine the clinical and demographic factors associated with attrition.

## Methods

### Design

This is a retrospective cohort study using routine program data.

### Study setting

CHD is located in the southern part of Maputo City, Mozambique, and has a total population of 329,872 inhabitants. The CRAM is an intermediary-level health facility that has four outpatient consultation rooms, a three-bed day-care unit, and a Kaposi sarcoma unit. CRAM serves as the only referral center for complicated HIV cases out of the five primary health centers of the CHD. Patients who fulfill the referral criteria (see Box 1) are received at CRAM and cared for until their condition becomes stable (defined as having a CD4 count of more than 200 cells/µl and showing no signs of therapeutic failure, adherence problems, or opportunistic infections [OIs] for more than 6 months). The patients are then referred back to their original health center with a medical summary that contains all the relevant information regarding patient treatment and care received at CRAM. Patients requiring admission and intensive care are immediately referred to tertiary care hospitals.

BOX 1 **Criteria for referral of patients from PHC to CRAM referral center in Maputo, Mozambique**

- Patients with CD4 less than 50 cells/µl and with signs of severe OI
- Patients in suspicion of ART first-line treatment failure
- Pediatric complex cases living with HIV
- Patients with Kaposi sarcoma eligible for chemotherapy
- Co-infection of HIV-1 with human T-lymphotropic virus (HTLV) or hepatitis B/C and HIV-2
- Patients suffering from side effects of ART treatment, including lactic acidosis
- Patients in critical health condition, lacking complementary examinations, and in need of more specialized care or invasive medical procedures.

PHC, primary health care; CRAM, Referral Centre of Alto Mae, Maputo City, Mozambique; OI, opportunistic infection; ART, antiretroviral therapy.

CRAM provides a comprehensive package of medical, laboratory, and psychological care, including second- and third-line ART for adults and children, chemotherapy for patients living with HIV and Kaposi sarcoma, and the diagnosis and treatment of OIs, side effects of ART drugs, psychiatric diseases, and other conditions. The center also provides psychosocial support, which includes counseling to enhance adherence, special psychological support for patients presenting with Kaposi sarcoma, and psychological care for those patients in need. A system for tracing those LTFU through phone calls and direct home visits has also been established. At the end of 2012, about 1,470 patients were enrolled on care, including more than 400 Kaposi sarcoma patients and around 250 patients in second line ART, making the CRAM one of the most important treatment centers for Kaposi sarcoma and second-line ART care in the country. During the study period, the Mozambican national guidelines (10) specified the eligibility criteria for ART initiation as fulfilling at least one of the following: 1) WHO stage 4, 2) WHO stage 3 and CD4 count <350 cells/µl, or 3) CD4 count<250 cells/µl irrespective of WHO stage.

### Study population

The study population includes all patients living with HIV who enrolled in the CRAM from January 1, 2009, to December 31, 2011. The study was conducted during March–November 2013. In this study, patients were followed till 31 March 2013 (the censor date).

### Data collection and analysis

The variables related to the study objectives, including age, sex, baseline body mass index (BMI; defined as the individual's body weight in kilograms divided by the square of their height in meters [kg/m^2^]), WHO clinical staging, Kaposi sarcoma (clinical diagnosis), ART status at time of presentation, ART regimen, year of enrollment, attrition (defined as discontinuation from care for any reason, including death, LTFU, and stopping ARV medications), and dates of enrollment and attrition, were extracted from the program's electronic database (FUCHIA; Epicentre, Paris, France) and exported for analysis. LTFU was defined as patients who did not return to the facility for a period of 60 days or more after their last scheduled appointment.

We used EpiData Analysis (version 2.2.2.182; EpiData Association, Odense, Denmark) and STATA (version 12.1; STATA Corp., College Station, TX, USA) for univariate and multivariate analysis, respectively. Time-to-event analysis was performed. Time was measured from the date of enrollment in CRAM to the date of attrition or censor date, whichever was earlier. We calculated attrition rates (death rates and LTFU rates) by adding the patients who discontinued care and dividing by the total number of person-years (PYs) of observation, expressed as rates per 100 PYs along with 95% confidence intervals (95% CI). To examine associations of attrition with demographic and clinical characteristics, hazard ratios with 95% confidence intervals were calculated. Kaplan–Meier survival curves (showing a cumulative incidence of attrition) were plotted to examine the differences across the strata of demographic and clinical factors. A log rank test was used to assess difference between the area under the curves, and a *P*-value less than 0.05 was considered statistically significant. Life tables were computed to calculate cumulative incidence of attrition at 1, 3, 6, and 12 months and 2, 3, and 4 years after enrollment. All the variables found to be statistically significant during bivariate analysis were used for a multivariate analysis using a Cox proportional hazard regression model, and adjusted hazard ratios (aHRs) were calculated to assess the independent effects of each variable after controlling for the confounding effect of other variables. The proportional hazard assumption was assessed using the Schoenfeld and scaled Schoenfeld residuals and log–log plots.

### Ethics approval

The study was approved by the Mozambican Ethical Review Committee and has met the MSF's (Geneva, Switzerland) Ethics Review Board–approved criteria for analysis of routinely collected program data. It also satisfies the requirements of the Ethics Advisory Group of the International Union Against Tuberculosis and Lung Disease (Paris, France) and has their approval.

## Results

A total of 1,657 patients were enrolled in care during the study period. Of them, 847 (51%) were men, and the mean (standard deviation) age was 36 (SD: 11) years. Table 1 shows the demographic and clinical characteristics of the individuals included in this analysis. The main reasons for referral to CRAM were that the patients were in advanced clinical stages (WHO stages 3 or 4, or less than 50 CD4 cells/µl) in 70% (1,158) of the cases, and 19% (314) had Kaposi sarcoma. Nearly one-third were severely malnourished. Data on CD4 counts were missing in nearly three-fourths of patients. Among those with known CD4 counts, the median was 27 cells/µl (interquartile range: 11–44). Nearly 85% of all the patients were not on ART at the time of presentation and were initiated on ART at CRAM. Among them, nearly 96% were started on a first-line ART regimen, while the rest were put on second-line or alternate first-line ART.

**Table 1:** Attrition rates by demographic and clinical characteristics among clients living with HIV and enrolled for care in Maputo, Mozambique, 2009–2011

Category	Subcategory	Number (%)	Attrition rate per 100 PY	(95% CI)
Total	–	1,657 (100)	28	(26–30)
Sex	Male	847 (51)	32	(29–36)
	Female	808 (49)	24	(22–27)
	Unknown	2 (0)	29	(4–208)
Age (years)	<25	143 (9)	30	(24–38)
	25–34	730 (44)	28	(25–31)
	35–44	414 (25)	25	(22–30)
	≥45	361 (22)	32	(28–37)
	Unknown	9 (1)	21	(8–57)
Kaposi sarcoma	Yes	314 (19)	41	(35–48)
	No	1,343 (81)	27	(25–29)
WHO stage	1	249 (15)	19	(15–24)
	2	257 (16)	17	(14–21)
	3	557 (34)	25	(22–28)
	4	584 (35)	46	(41–51)
	Not recorded	10 (1)	29	(12–71)
CD4 count	<50	346 (21)	15	(12–17)
	50–249	40 (2)	14	(8–25)
	250–349	13 (1)	34	(17–69)
	>350	16 (1)	25	(13–49)
	Unknown	1,242 (75)	36	(33–39)
BMI (kg/m^2^)	<16	186 (11)	45	(38–54)
	16–18.5	334 (20)	30	(26–35)
	≥18.5	939 (57)	21	(19–23)
	Unknown	198 (12)	61	(52–73)
ART status at the time of presentation	Yes	247 (15)	627	(553–712)
	No	1,410 (85)	20	(19–22)
ART regimen*	First-line	1,356 (96)	20	(19–22)
	Second-line	54 (4)	17	(10–27)
Year of admission	2009	590 (36)	19	(17–21)
	2010	407 (25)	28	(24–33)
	2011	660 (40)	47	(42–53)

100 PYs, 100 person-years of observation; CI, confidence interval; WHO, World Health Organization; BMI, body mass index; ART, antiretroviral therapy.

* Only for those in whom ART was initiated at the study site and information was available.

The attrition rates among the study population are shown in Table 2. The patients contributed to a total observation period of 2,762 PYs, during which 793 attrition events occurred: 137 deaths and 656 LTFU. Thus, the overall attrition rate was 28.7 per 100 PYs, where the mortality rate contributes 5.0 (95% CI: 4.2–5.9) per 100 PYs and LTFU rate contributes 23.7 (95% CI: 21.9–25.6) per 100 PYs. Attrition rates were significantly higher among men, those with Kaposi sarcoma, people in WHO clinical stage 3 or 4, those with low BMI, and people enrolled in care during the year 2010 and 2011. The highest attrition rates were observed among those who were already on ART at the time of presentation to the clinic.

**Table 2:** Cumulative incidence (in %) of attrition among clients living with HIV and enrolled for care in Maputo, Mozambique, 2009–2011 (*N*=1,657)

Period	Cumulative number of attritions (*N*)	Number of deaths	Number of LTFU	Cumulative incidence of attrition (95% CI)
1 month	298	38	260	18 (16–20)
3 months	433	77	356	26 (24–28)
6 months	522	97	425	32 (29–34)
1 year	615	110	505	37 (35–40)
2 years	731	125	606	45 (43–48)
3 years	781	134	647	47 (45–50)
4 years	793	137	656	48 (45–50)

LTFU, lost to follow up; CI, confidence interval.

The cumulative incidence of attrition and its timing are shown in Table 2. It is to be noted that nearly 77% of all attrition happened within the first year of enrollment, and of the attrition during the first year, about 50% occurred during the first month – this was similar for deaths and LTFU.

**Fig. 1:**
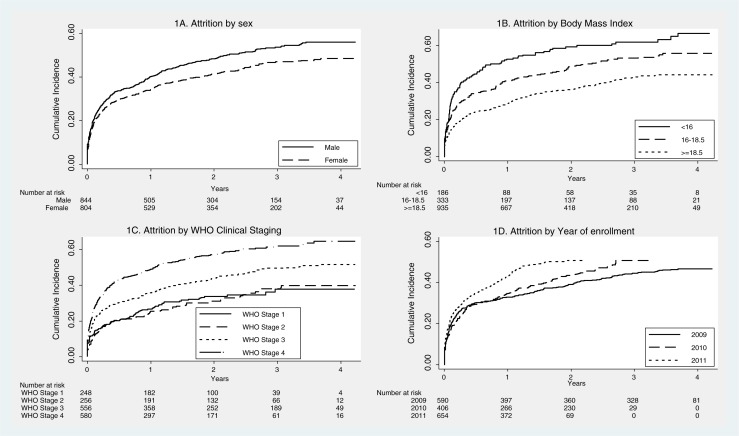
Kaplan–Meier survival plots showing cumulative incidence of attrition by sex (1A), body mass index (1B), World Health Organization Clinical Staging (1C), and year of enrollment (1D) among clients living with HIV and enrolled for care in Maputo, Mozambique, 2009–2011.

**Fig. 2:**
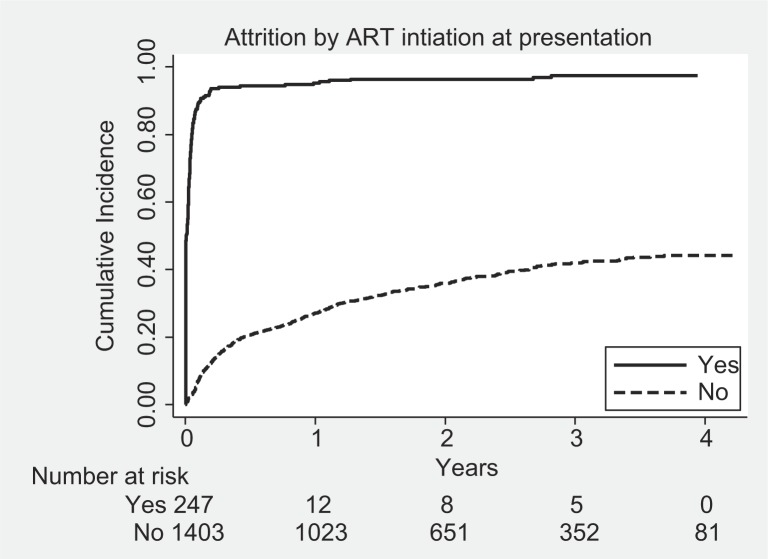
Kaplan–Meier survival plots showing cumulative incidence of attrition by antiretroviral therapy status at presentation, among clients living with HIV and enrolled for care in Maputo, Mozambique, 2009–2011.

Since most of the attrition was contributed by LTFU patients and previous studies have shown that nearly 50% of LTFU patients actually died, we decided to examine overall attrition rates as the outcome variable. Table 3 shows the Cox proportional hazard regression analysis of factors associated with attrition. The factors independently associated with attrition after simultaneous adjustment of all confounders were male sex, low BMI, WHO clinical stage 3 or 4, and later year of enrollment (Fig. 1), and ‘being already on ART’ at the time of presentation to the clinic was the most significant factor (Fig. 2).

**Table 3:** Clinical and demographic factors associated with attrition among clients living with HIV and enrolled for care in Maputo, Mozambique, 2009–2011

Category	Subcategory	Unadjusted HR (95% CI)	Adjusted HR (95% CI)
Sex	Male	**1.23 (1.0–1.4)**	**1.15 (1.0–1.3)**
	Female	1	1
Kaposi sarcoma	Yes	**1.26 (1.0–1.5)**	1.01 (0.7–1.2)
	No	**1**	1
WHO stage	1	1	1
	2	0.97 (0.7–1.3)	1.23 (0.9–1.6)
	3	**1.45 (1.1–1.8)**	**1.30 (1.0–1.6)**
	4	**2.10 (1.6–2.6)**	**1.91 (1.4–2.5)**
BMI (kg/m^2^)	<16	**1.91 (1.5–2.3)**	**1.51 (1.2–1.8)**
	16–18.5	**1.42 (1.1–1.7)**	**1.51 (1.2–1.8)**
	≥18.5	1	1
ART status at presentation	Yes	**13.96 (11.7–16.5)**	**13.71 (11.4–16.4)**
	No	1	1
ART regimen	First line	1	NA
	Second line	0.74 (0.4–1.2)	
Year of admission	2009	1	1
	2010	1.14 (0.9–1.3)	**1.29 (1.0–1.5)**
	2011	**1.43 (1.2–1.6)**	**1.61 (1.3–1.9)**

HR, hazard ratio; CI, confidence interval; WHO, World Health Organization; BMI, body mass index; ART, antiretroviral therapy.

Hazard ratios in bold font are statistically significant (*P* value less than 0.05).

## Discussion

To our knowledge, this is one of the first analyses of attrition from care focused on patients living with HIV and with advanced disease who were enrolled in an intermediary-level health facility. Overall, nearly 50% of all patients enrolled in care in our facility either died or were LTFU during the observation period of 4 years. Among these, nearly 80% of the attrition happened during the first year. Key factors associated to attrition were male sex, WHO clinical stage 3 or 4, low BMI, year of enrollment 2010 and 2011, and ART status at the time of presentation to the clinic.

This analysis describes a worrisome high attrition rate within the first year of care, with a considerable proportion of the events occurring during the first month after enrollment in care. This is one of the highest attrition rates reported from Mozambique as compared to previous studies, which reported an attrition rate from 21% up to 26% (6, 11). Unlike previous studies from Mozambique, which were conducted among all patients living with HIV, this study focused on patients living with HIV in advanced stages and with severe complications, which could partly explain the high attrition. While the exact reasons are not clear, we discuss a few based on our experience of working in the clinic.

First, the clinical and immunological profile of patients in the center indicates that those who were in the very advanced stages of their disease had a high risk of mortality. Nearly 85% of the patients who visited the facility were not initiated on ART at the time of presentation and had a median CD4 count of 27 cells/µl, indicating delays in diagnosis, enrollment into HIV care, and initiation of ART despite enrollment. Studies have shown that nearly 50% of all people living with HIV do not know their HIV status (12). Given the generalized HIV epidemic in Mozambique, innovative strategies to increase HIV test uptake, including community-based HIV testing using point-of-care HIV tests, are urgently required, followed by early entry into HIV care. Currently, ART programs do not monitor pre-ART attrition, but systematically providing attention on reducing pre-ART attrition may prevent late presentations to care. Further, in order to reduce the number of patients initiating ART at advanced disease stages, there is a need to adopt the new WHO 2013 ART guidelines, which recommend early initiation of ART and change the threshold of ART initiation from a CD4 count of 350 cells/µl to 500 cells/µl.

Second, the highest attrition rates were found among those who were already receiving ART from elsewhere at the time of presentation to the referral center. Nearly three-fourths of them had advanced clinical disease, and their attrition could in our study possibly represent unrecorded deaths as has been showed by other studies (13, 14). However, it is also possible that some of them returned back to their original primary health facilities for continuing HIV care or sought care at alternative sources of ART, and hence represent ‘undocumented transfers’ from the referral clinic. This could be overestimating the attrition rates observed in the study. Better documentation of transfer out and deaths and further investigation using qualitative methods are required to understand the exact reasons of attrition in the first month (15).

Third, our analysis showed that a number of individual-level factors were independently associated with attrition and merit special attention. Like in other studies in Mozambique, in our multivariate analysis male sex was associated with high attrition rates (11). Reasons for this are unknown, but could be related to late presentation to care, poor treatment adherence, and poor healthcare-seeking behavior as reported in previous studies (16). Specific interventions to reach men earlier could include HIV-testing campaigns in the community, workplaces, or sporting events.

Fourth, people who were malnourished were at a higher risk of attrition, as has been reported from other ART programs in sub-Saharan Africa. A recent study from Mozambique examining clinic site-related factors associated with attrition has shown that ART centers that offered nutritional support services had better rates of enrollment and retention (17). Highlighting the importance of nutritional support among patients living with HIV attending our clinic and including a tailored nutritional support in the package of care offered in the health facility could contribute to improving the outcomes of the program. Also, poor nutritional status with subsequent deterioration of the immunological profile of the patients living with HIV could predispose for severe OIs, with tuberculosis (TB) being one of the most common presentations. The burden of TB during early HIV care is particularly heavy in sub-Saharan African settings, where this presents a major challenge in treatment programs. An important proportion of patients living with HIV and with asymptomatic TB (or TB with minimal symptoms) are not clinically recognized until immune recovery triggers the development of symptoms during early ART (18, 19). Innovative strategies for intensive investigations of OI, which include intensified TB case finding, the use of new diagnostics tools like the Xpert MTB/RIF assay or the new assay that detects mycobacterial lipoarabinomannan (LAM) antigen present in urine, and presumptive or empirical TB treatment prior to starting ART, should be considered in this setting in order to develop a more integrated healthcare delivery for TB, HIV, and other OIs (20).

Finally, the patients enrolled in care in 2010 and 2011 had higher attrition rates than in 2009. This could possibly be related to strict implementation of referral criteria in July 2010, thus resulting in a situation wherein only patients living with HIV who experience complications are enrolled into care.

Our analysis has several strengths. First, we used routinely collected program data, and hence the findings reflect the realities in the field. Second, as mentioned earlier, this study provides information specifically on severely immune-compromised patients living with HIV who were enrolled in an intermediary-level health facility, whereas most other reports were focused mainly on overall ART programs. Third, we followed the strengthening the reporting of observational studies in epidemiology (STROBE, (21) guidelines for reporting of observational studies, including ethics.

There were a few limitations mainly related to the operational nature of the study. First, data were missing in a substantial proportion of patients on key variables like CD4 count at admission and BMI. The lack of CD4 count could be partially explained by the limited access to CD4 count testing during the study period in CHD, with the CD4 testing facility available only in the laboratory of the Maputo Central Hospital. In addition, the laxity in documentation of CD4 data in the electronic register or the medical files may have contributed to the missing data. Urgent measures are required, including sensitizing the clinic staff about the importance of recording and reporting and strengthened supervision and monitoring. Second, we may be overestimating attrition. As mentioned earlier, we could not document the exact final outcomes of LTFU. It is possible that many of these could be on HIV care from other facilities and hence are poorly documented. Third, we could not identify the exact reason of attrition. Future research, including qualitative methods (with in-depth patient interviews), is needed for a better understanding of the reasons for attrition. Since most of the patients have access to a mobile phone, this could be used not only for tracking of LTFU cases, but also to reinforce messages and prevent LTFU.

## Conclusions

We identified that overall attrition rates among patients living with HIV and enrolled into care at the intermediary referral center in Maputo were high, and were mainly related to advanced stage of clinical disease. Urgent measures are required to address this, including developing innovative ways of increasing HIV test uptake, adopting the new WHO 2013 criteria for ART initiation, providing nutritional supplementation in the package of care, and providing special attention to men and those who are already on ART elsewhere at the time of presentation to the clinic. Qualitative research is required to understand the exact reasons of LTFU and to design informed evidence-based interventions.
